# Addition of Dulaglutide or Empagliflozin to Standard-of-Care Treatment: Effect on Liver Steatosis in Patients With Type 2 Diabetes Mellitus

**DOI:** 10.7759/cureus.53813

**Published:** 2024-02-07

**Authors:** Emmanouil Koullias, Maria Papavdi, Stavros Athanasopoulos, Asimina Mitrakou, Melanie Deutsch, Pavlos Zoumpoulis, Emmanuel Manesis, Anastasia Thanopoulou, John Koskinas

**Affiliations:** 1 2nd Department of Internal Medicine, Medical School, National and Kapodistrian University of Athens, Hippocration General Hospital, Athens, GRC; 2 2nd Department of Internal Medicine, Medical School, Hippocration General Hospital, National and Kapodistrian University of Athens, Athens, GRC; 3 Department of Therapeutic, Medical School, Alexandra General Hospital, National and Kapodistrian University of Athens, Athens, GRC; 4 2nd Department of Internal Medicine, Medical School, Hippocration General Hospital, National and Kapodistrian University of Athens, Athens, GRC; 5 Department of Diagnostic Echotomography, Echomed, Athens, GRC; 6 Department of Internal Medicine, Euroclinic, Athens, GRC

**Keywords:** magnetic resonance imaging-proton density fat fraction, empagliflozin, dulaglutide, type 2 diabetes mellitus (type 2 dm), nafld and fatty liver and steatosis

## Abstract

Background

Patients with liver steatosis and diabetes mellitus can benefit from medications like glucagon-like peptide 1 receptor agonists or sodium-glucose co-transporter 2 inhibitors, as far as both hyperglycemia and fatty liver are concerned. Studies comparing members of both these families have not yet been published. We aimed to compare the effects of Empagliflozin and Dulaglutide, focusing primarily on liver steatosis.

Methodology

This prospective, observational, controlled study enrolled 78 patients from two centers in Athens, Greece. Adults with type 2 diabetes mellitus (DM2) and nonalcoholic fatty liver disease were assigned to one of three groups and received either Empagliflozin or Dulaglutide or any other medical treatment deemed appropriate by their physician. The primary endpoint was the reduction in liver fat fraction, assessed using magnetic resonance imaging-proton density fat fraction. Additionally, we evaluated the proportion of patients achieving a relative reduction above 30% of their initial liver fat concentration.

Results

The Empagliflozin group exhibited a reduction in liver fat fraction. Furthermore, the percentage of patients with a relative reduction of liver steatosis, >30%, was significantly larger in this group, compared to the Dulaglutide and Control groups. Significant body weight reduction was observed in all three groups, but no improvement in fibrosis assessing scores was noted.

Conclusions

Empagliflozin is effective in improving liver steatosis, while Dulaglutide does not exhibit a similar effect. Larger studies, comparing these or related agents, are necessary, to further assess benefits in patients with DM2 and nonalcoholic fatty liver.

## Introduction

Nonalcoholic fatty liver (NAFL) disease is the most common condition among hepatopathies worldwide. The spectrum of the disease ranges from plain NAFL, due to triglyceride accumulation, to nonalcoholic steatohepatitis (NASH), due to inflammation and fibrosis, and liver cirrhosis. Prevalence among patients with type 2 diabetes mellitus (DM2) is very high, while the coexistence of these two entities is known to lead to faster progression of liver disease than NAFL alone [1].

Current clinical practice guidelines for liver steatosis focus on weight loss, through exercise, diet, and general lifestyle modifications, while a specific medical treatment has not been incorporated. Older medicines, such as metformin or the dipeptidyl peptidase-4 inhibitors (DPP-4is) have not been shown to exhibit any important benefits, regarding NASH or NAFL [1,2]. However, several studies aimed to investigate the effectiveness of newer agents, such as glucagon-like peptide 1 receptor agonists (GLP1-ras) or sodium-glucose co-transporter 2 inhibitors (SGLT2-is), regarding beneficial effects on NAFL disease (NAFLD). Liraglutide and semaglutide have shown encouraging results in ameliorating steatohepatitis and inducing weight loss, irrespective of the presence of DM2 [3-6]. Relevant research regarding Dulaglutide, another member of the GLP1-ras family, is not as rich [7,8], with only one randomized controlled trial (RCT) studying its effect on liver fat content reduction. Similarly, the SGLT2-is, with Empagliflozin foremost, seems to be useful as well [9-11]. Interestingly, both medication families appear to provide a protective effect concerning renal and cardiac functions [12-14].

However, head-to-head studies comparing members of both groups have not yet been published. Therefore, we designed this study to compare the effects of Empagliflozin and Dulaglutide on liver fat content, glycemic control, and weight loss in patients with DM2.

This paper was previously presented as an oral presentation at the 21st European Congress of Internal Medicine (ECIM) joined with the 12th International Congress of Internal Medicine on March 17, 2023.

## Materials and methods

Study design

This prospective, observational, open-label, parallel-group trial, with a control group, was conducted at two Diabetes Centers in Athens, Greece, from June 2018 to May 2021. The study was approved by the Medical Research and Scientific Protocols - Committee of the Scientific Council of the General Hospital of Athens “Hippocration” (protocol number SC12/16-6-2016) and was registered with the Medical School of the National and Kapodistrian University of Athens (protocol number 1516021038-28/03/16) and conformed to good clinical practice guidelines. Patient consent was given before inclusion in the study and after the patients had been prescribed appropriate treatment by their physicians at the corresponding diabetes center. Patients were monitored for one year after inclusion. The study has also been registered with the ClinicalTrials.gov Protocol Registration and Results System (registration number NCT05946148).

Patient sample

Patients aged 25 to 75 years with DM2 and NAFLD were eligible if they had been on a stable DM2 treatment regimen for the past six months. Exclusion criteria included a recent (last five years) medical history of cancer, pancreatitis, viral hepatitis, or any other cause of liver disease (alcohol abuse, autoimmune hepatitis, hemochromatosis, heart failure, etc.), except for NAFLD. In addition, patients under corticotherapy were excluded, along with those scheduled for bariatric surgery or planning for pregnancy.

Procedures

Patients were assigned by their treating physicians to receive either Dulaglutide or Empagliflozin as an add-on to their previous treatment regimen. Alternatively, they were included in the control group, where optimal treatment (excluding agents of the GLP1-ras or SGLT2-is families) was prescribed, focusing on glycemic control. Patients receiving Pioglitazone were not included in the study. The monitoring duration was determined to be 52 weeks. All patients received proper dietary and exercise advice, as well as proper training regarding their treatment plan. Patients had an intermediate visit at 26 weeks, and additional visits were conducted if deemed necessary on a case-by-case basis.

Assessments

All patients underwent a thorough clinical examination at the time of inclusion, at 26 weeks, and at 52 weeks following treatment initiation. Furthermore, all patients underwent ultrasound and shearwave elastography, both performed by a blinded radiologist. Additionally, extended blood tests were conducted at the same intervals, encompassing assessments for viral hepatitis, fasting blood glucose, glycated hemoglobin A1c (HbA1c), liver enzymes, lipids, urea, creatinine, and a full blood count. The collected data were used to calculate the fatty liver index (FLI), the Fibrosis-4 index (FIB-4), the aspartate aminotransferase-to-platelet ratio index (APRI), and the NAFLD fibrosis score (NFS), at each time point. The FLI score was calculated using the following equation, by Bedogni et al. [15]: {FLI = (e^0.953×ln (TG) + 0.139*BMI [kg/m2] + 0.718*ln (GGT [IU/L]) + 0.053*Waist Circumference [cm] − 15.745^) / (1+e^0.953*ln (TG [mg/dL]) + 0.139*BMI [kg/m2] + 0.718*ln (GGT [IU/L]) + 0.053*Waist Circumference [cm] − 15.745)^ *100}. The FIB-4 Index was calculated using the following equation, by Sterling et al. [16]: {FIB-4= (Age[years]*AST[IU/L])/(Platelets [10^9^/L]*√ALT[IU/L])}. The APRI was calculated using the following equation, by Wai et al [17]: {APRI = (AST [IU/L]) / (AST Upper Limit of Normal [IU/L]) / (Platelets [10^9^/L])} and the NFS was calculated using the following equation, by Angulo et al [18]: {NFS=-1.675+(0.037*Age[years]) + (0.094*BMI [kg/m2]) + (1.13*IFG/Diabetes [yes = 1, no = 0]) + (0.99*AST/ALT ratio) - (0.013*Platelets [10^9^/L]) - (0.66*Albumin [g/dl])}. Finally, the patients were submitted to a magnetic resonance imaging-proton density fat-fraction (MRI-PDFF) scan, to quantify steatosis, at the time of inclusion and at 52 weeks after treatment initiation. During the evaluation of liver steatosis, focal liver lesions, vessels, and the bile duct were avoided. All scans were analyzed by the same radiologist, who was blinded to treatment allocation, as well as clinical data. NAFLD was defined as an MRI-PDFF-derived assessment of liver fat fraction (LFF) >5%.

Endpoints

The primary endpoints of the study were the change of LFF after 52 weeks of treatment, as well as the achievement of >30% relative reduction of LFF. Secondary endpoints included evaluation of glycemia, body mass index (BMI), and various scores estimating inflammation, steatosis, and fibrosis (FLI, FIB-4, APRI, and NFS).

Statistical analyses

Based on previous studies [4], we estimated that at least 22 patients for each of the three groups would be necessary. The significance level for all statistical tests was set at 5%. In the end, at least 25 patients were included in each group. All patients were of Caucasian origin. Patients who did not undergo an end-of-treatment evaluation of a particular parameter were classified as stable relative to their last follow-up. Statistical analyses were performed with IBM SPSS Statistics for Windows Version 25 (IBM Corp., Armonk, NY). Endpoints were compared among the three treatment groups using the Kruskal-Wallis test, while a comparison between screening and 52-week follow-up was performed with the Wilcoxon signed ranks test for each group. Comparisons between any two groups were conducted using the Mann-Whitney U test.

## Results

Of the 90 patients screened between June 2018 and December 2020, 78 were enrolled and completed the follow-up at 52 weeks (per-protocol population), of which 28 were in the control group and 25 in each of the Dulaglutide and Empagliflozin groups. The other 12 patients were either lost to follow-up (six) or excluded due to protocol violations (five), or adverse events (one). In the per-protocol population, out of 78 patients, 56 (71.8%) were male and 22 (28.2%) were female. At baseline, mean BMI (± SEM) was 29.64 (± 0.91) kg/m^2^ in the Empagliflozin group, 35.05 (± 1.11) kg/m^2^ in the Dulaglutide group, and 31.53 (± 1.00) kg/m^2^ in the control group. Additionally, the mean age (± SEM) was 63.24 ± 1.45, 54.36 ± 2.49, and 55.00 ± 2.38 years and the mean LFF (± SEM) of participants was 12.31 ± 1.76%, 19.51 ± 1.91%, and 13.91 ± 1.50%, in the Empagliflozin, Dulaglutide, and Control groups, respectively (Table 1).

**Table 1:** Baseline characteristics of the per-protocol study population. Values are presented as mean (±SEM). ^*^P < 0.05. ALT, alanine transaminase; AST, aspartate transaminase; BMI, body mass index; C, Control; D, Dulaglutide; E, Empagliflozin; FIB-4, Fibrosis 4; FLI, fatty liver index; FPG, fasting plasma glucose; HbA1c, glycated hemoglobin A1c; HDL, high-density lipoprotein; HC, hip circumference; LDL, low-density lipoprotein; MRI-PDFF, magnetic resonance imaging-proton density fat fraction; NFS, NAFLD fibrosis score; SEM, standard error of the mean; SWE, shearwave elastography; TC, total cholesterol; TG, triglyceride; WC, waist circumference

Characteristics	Empagliflozin (E)	Dulaglutide (D)	Control (C)	*P* (between groups)
*n* (male/female)	25 (18/7)	25 (21/4)	28 (17/11)	-
Age (years)	63.24 ± 1.45	54.36 ± 2.49	55.00 ± 2.38	0.011* (E older than D and C)
Weight (kg)	85.16 ± 3.56	102.66 ± 4.02	93.41 ± 3.75	0.004* (E lower than D and C)
BMI (kg/m^2^)	29.64 ± 0.91	35.05 ± 1.11	31.53 ± 1.00	0.002* (E and C lower than D)
WC (cm) - Men	103.8 ± 3.1	120.0 ± 3.1	112.8 ± 3.4	0.003* (E smaller than D)
WC (cm) - Women	107.4 ± 3.1	113.5 ± 4.1	98.5 ± 8.3	0.271
HC (cm) - Men	104.6 ± 2.5	113.0 ± 2.1	109.8 ± 1.6	0.004* (E smaller than D and C)
HC (cm) - Women	109.1 ± 4.2	116.8 ± 4.7	115.0 ± 7.0	0.669
AST (U/L)	30.1 ± 3.2	24.7 ± 1.7	27.1 ± 2.4	0.944
ALT (U/L)	39.9 ± 4.0	38.7 ± 3.5	40.1 ± 6.7	0.943
FPG (mg/dL)	131.5 ± 9.3	135.3 ± 13.3	110.5 ± 4.5	0.446
HbA1c (%)	7.23 ± 0.21	7.29 ± 0.26	6.40 ± 0.14	0.005* (C lower than E and D)
TC (mg/dL)	142.0 ± 7.1	161.2 ± 7.0	173.8 ± 8.1	0.016* (E lower than C)
TG (mg/dL)	134.8 ± 10.8	158.9 ± 14.1	129.6 ± 11.5	0.137
LDL (mg/dL)	74.4 ± 6.1	88.7 ± 6.5	101.9 ± 7.1	0.474
HDL (mg/dL)	40.7 ± 1.5	39.7 ± 1.6	43.5 ± 1.9	0.384
SWE stiffness (kPa)	7.1 ± 0.9	6.1 ± 0.3	6.6 ± 0.7	0.736
APRI	0.410 ± 0.056	0.279 ± 0.019	0.374 ± 0.054	0.189
FIB-4 Index	1.370 ± 0.131	0.864 ± 0.069	1.206 ± 0.200	0.003* (E higher than D and C)
NFS	-0.706 ± 0.143	-1.045 ± 0.253	-0.859 ± 0.235	0.863
FLI	66.10 ± 4.74	87.98 ± 2.67	71.33 ± 4.98	0.002* (E, C lower than D)
MRI-PDFF (%)	12.31 ± 1.76	19.51 ± 1.91	13.91 ± 1.50	0.004* (E, C lower than D)

The majority of the patients in the Dulaglutide group were obese, while cardiovascular disease was more prevalent in the Empagliflozin group (Table 2).

**Table 2:** Comorbidities and concomitant drug use. ^*^Hypertension was defined as prior treatment with a blood pressure-lowering agent or a measurement of >140/90 mmHg at screening. ^**^Dyslipidemia was defined as prior treatment with a lipid-lowering drug or having a detected high LDL level at screening. DPP-4, dipeptidyl peptidase-4; BMI, body mass index; med, medication; LDL, low-density lipoprotein

Comorbidities	Empagliflozin (*n *= 25)	Dulaglutide (*n *= 25)	Control (*n *= 28)
Hypertension*	18/25 (72%)	17/25 (68%)	18/28 (64.3%)
Dyslipidemia**	22/25 (88%)	21/25 (84%)	18/28 (64.3%)
Obesity (BMI > 30 kg/m^2^)	10/25 (40%)	21/25 (84%)	16/28 (57.1%)
Hypothyroidism	4/25 (16%)	4/25 (16%)	8/28 (28.6%)
Cardiovascular disease	11/25 (44%)	4/25 (16%)	0/28 (0%)
Chronic kidney disease	3/25 (12%)	0/25 (0%)	0/28 (0%)
Smoking	19/25 (76%)	18/25 (72%)	12/28 (42.9%)
Concomitant drug use	Empagliflozin (*n *= 25)	Dulaglutide (*n *= 25)	Control (*n *= 28)
Metfomin	25/25 (100%)	22/25 (88%)	28/28 (100%)
DPP-4 inhibitors	6/25 (24%)	0/25 (0%)	4/28 (14.3%)
Sulfonylureas	2/25 (8%)	0/25 (0%)	0/28 (0%)
Thiazolidinediones	0/25 (0%)	0/25 (0%)	0/28 (0%)
Insulin	4/25 (16%)	6/25 (24%)	2/28 (7.1%)
Antihypertensive med	18/25 (72%)	16/25 (64%)	15/28 (53.6%)
Lipid-lowering med	24/25 (96%)	20/25 (80%)	16/28 (57.1%)
Antiplatelet med	12/25 (48%)	9/25 (36%)	3/28 10.7%)

Liver fat fraction

In the Empagliflozin group, MRI-PDFF exhibited a significant decrease between baseline and week 52 (from 12.31 ± 1.76% to 9.34 ± 1.39%, *P *= 0.007). On the contrary, no statistical improvement was observed in the Dulaglutide and Control groups. Comparisons between groups, utilizing the Mann-Whitney U test, revealed that the improvement in MRI-PDFF was significantly greater in the Empagliflozin group compared to the Control group (-2.63 vs. -0.16, *U *= 214.00, *z *= -2.423, *P *= 0.015). A similar trend was observed between the Empagliflozin and Dulaglutide groups (-2.63 vs. -0.60, *U *= 231.00, *z *= -1.581, *P *= 0.114), favoring Empagliflozin. When a cutoff was set at a 30% reduction of liver fat content, we observed improvement in 13 out of 25 patients in the Empagliflozin group, compared to three in the Dulaglutide group and two in the Control group. In this context, the Empagliflozin group demonstrated superior results compared to the Dulaglutide group (*P* = 0.003) and the Control group (*P* = 0.000).

BMI, HbA1c, and FLI score

A significant decrease was observed in BMI (± SEM) in all three groups (Empagliflozin: -1.79 ± 0.40, *P *= 0.000; Dulaglutide: -1.72 ± 0.52, *P *= 0.003; Control: -0.71 ± 0.30, *P *= 0.025). Similarly, a significant improvement in the FLI score was detected (Empagliflozin: -7.73 ± 2.65, *P* = 0.008; Dulaglutide: -5.49 ± 2.29, *P* = 0.040; Control: -5.46 ± 2.54, *P* = 0.041), with no significant difference between groups (*P *= 0.189). A Kruskal-Wallis test exhibited the presence of statistically significant differences between groups, regarding BMI (*P* = 0.049). A Mann-Whitney U test showed a significant difference between Empagliflozin and Control groups (*P *= 0.010), but not between Dulaglutide and Control groups (*P *= 0.142), despite Dulaglutide group having a tendency for greater weight loss. There was no significant difference between Empagliflozin and Dulaglutide (*P* = 0.705) in terms of BMI reduction. As far as HbA1c improvement was concerned, no significant difference was observed between groups. However, significant reduction was observed in the Empagliflozin group (Empagliflozin: -0.447 ± 0.165, *P* = 0.012; Dulaglutide: -0.329 ± 0.189, *P *= 0.094; Control: -0.115 ± 0.121, *P *= 0.350). The lack of impressive results, especially concerning HbA1c, could be attributed partly to the already relatively well-controlled glycemia of most participants prior to inclusion in the study. Detailed results are shown in Table 3.

**Table 3:** Changes in endpoints between baseline and follow-up at 52 weeks in the three groups. Values are presented as mean ± SEM. ^*^P < 0.05 in bold. ALT, alanine transaminase; AST, aspartate transaminase; APRI, AST-to-platelet ratio index; BMI, body mass index; BT, before treatment; FIB-4, Fibrosis-4; FLI, fatty liver index; FPG, fasting plasma glucose; FU, follow-up; HbA1c, glycated hemoglobin A1c; HDL, high-density lipoprotein; LDL, low-density lipoprotein; M/F, male/female; MRI-PDFF, magnetic resonance imaging-proton density fat fraction; NFS, NAFLD fibrosis score; SEM, standard error of the mean; SWE, shearwave elastography; TC, total cholesterol; TG, triglyceride

Characteristics	Empagliflozin			Dulaglutide			Control		
*n* (M/F)	*n *= 25 (18/7)			*n *= 25 (21/4)			*n *= 28 (17/11)		
	BT	FU at 52 weeks	Change (*P*)	BT	FU at 52 weeks	Change (*P*)	BT	FU at 52 weeks	Change (*P*)
Weight (kg)	85.16 ± 3.56	79.88 ± 2.76	-5.28 ± 1.26 (0.001)*	102.66 ± 4.02	97.97 ± 3.67	-4.69 ± 1.51 (0.006)*	93.41 ± 3.75	91.30 ± 3.60	-2.04 ± 0.92 (0.031)*
BMI (kg/m^2^)	29.64 ± 0.91	27.85 ± 0.72	-1.79 ± 0.40 (0.000)*	35.05 ± 1.11	33.33 ± 0.94	-1.72 ± 0.52 (0.003)*	31.53 ± 1.00	30.82 ± 0.94	-0.71 ± 0.30 (0.025)*
Waist (cm)	104.80 ± 2.40	100.82 ± 1.74	-3.98 ± 1.06 (0.001)*	120.05 ± 2.72	116.27 ± 2.65	-3.77 ± 1.20 (0.007)*	110.67 ± 3.77	108.17 ± 3.71	-2.50 ± 6.25 (0.024)*
Waist (cm) - Men	103.8 ± 3.1	99.7 ± 2.2	-4.1 ± 1.3 (0.007)*	120.8 ± 3.1	117.4 ± 3.0	-3.4 ± 1.3 (0.019)*	113.5 ± 3.4	110.5 ± 3.6	-3.0 ± 1.8 (0.055)
Waist (cm) - Women	107.4 ± 3.1	103.8 ± 2.1	-3.6 ± 1.8 (0.091)	115.5 ± 4.1	109.3 ± 2.4	-6.2 ± 3.6 (0.285)	106.3 ± 8.3	104.6 ± 8.0	-1.7 ± 0.9 (0.102)
Hips (cm)	105.84 ± 2.13	102.48 ± 1.46	-3.36 ± 0.90 (0.001)*	113.52 ± 1.88	111.64 ± 1.82	-1.89 ± 0.92 (0.066)	111.81 ± 2.81	109.61 ± 2.74	-2.19 ± 1.10 (0.061)
Hips (cm) - Men	104.6 ± 2.5	101.4 ± 1.7	-3.2 ± 1.1 (0.006)*	113.0 ± 2.1	110.9 ± 2.0	-2.1 ± 1.1 (0.080)	109.8 ± 1.6	107.7 ± 2.3	-2.0 ± 1.5 (0.191)
Hips (cm) - Women	109.1 ± 4.2	105.3 ± 2.6	-3.9 ± 1.7 (0.078)	116.8 ± 4.7	116.0 ± 4.7	-0.8 ± 0.6 (0.180)	115.0 ± 7.0	112.6 ± 6.2	-2.4 ± 1.7 (0.168)
AST (U/L)	30.16 ± 3.22	26.08 ± 2.66	-4.08 ± 3.44 (0.188)	24.72 ± 1.73	24.16 ± 1.66	-0.56 ± 1.90 (0.784)	27.14 ± 2.36	28.46 ± 2.80	1.32 ± 2.55 (0.313)
ALT (U/L)	39.88 ± 3.99	29.96 ± 3.28	-9.92 ± 4.00 (0.008)*	38.72 ± 3.47	34.28 ± 2.87	-4.44 ± 3.49 (0.294)	40.14 ± 6.70	34.71 ± 3.89	-5.43 ± 6.51 (0.915)
FPG (mg/dL)	131.48 ± 9.27	126.16 ± 8.90	-5.32 ± 9.97 (0.936)	135.28 ± 13.35	126.64 ± 11.61	-8.64 ± 12.74 (0.775)	110.54 ± 4.55	108.43 ± 3.84	-2.11 ± 4.75 (0.602)
HbA1c (%)	7.23 ± 0.21	6.79 ± 0.13	-0.447 ± 0.165 (0.012)*	7.29 ± 0.26	6.96 ± 0.20	-0.329 ± 0.189 (0.094)	6.40 ± 0.14	6.28 ± 0.10	-0.115 ± 0.121 (0.350)
TC (mg/dL)	142.04 ± 7.13	148.80 ± 8.98	6.76 ± 9.99 (0.412)	161.24 ± 7.02	165.64 ± 6.93	4.40 ± 4.86 (0.397)	173.79 ± 8.12	165.79 ± 6.55	-8.00 ± 6.42 (0.221)
TG (mg/dL)	134.76 ± 10.79	154.56 ± 19.39	19.80 ± 17.38 (0.346)	158.92 ± 14.06	169.40 ± 21.77	10.48 ± 18.58 (0.582)	129.57 ± 11.46	124.04 ± 12.80	-5.54 ± 8.25 (0.558)
LDL (mg/dL)	74.40 ± 6.06	74.68 ± 7.48	0.28 ± 8.12 (0.415)	88.68 ± 6.48	91.92 ± 7.17	3.24 ± 5.24 (0.557)	101.89 ± 7.08	96.21 ± 5.56	-5.68 ± 5.62 (0.312)
HDL (mg/dL)	40.72 ± 1.55	43.40 ± 1.70	2.68 ± 1.44 (0.075)	39.68 ± 1.59	40.84 ± 1.37	1.16 ± 1.24 (0.360)	43.50 ± 1.90	45.93 ± 1.73	2.43 ± 1.09 (0.034)*
SWE stiffness (kPa)	7.056 ± 0.940	6.960 ± 1.644	-0.096 ± 0.784 (0.046)*	6.096 ± 0.348	6.436 ± 0.327	0.340 ± 0.423 (0.201)	6.646 ± 0.675	6.929 ± 0.616	0.283 ± 0.214 (0.138)
APRI score	0.410 ± 0.056	0.415 ± 0.104	0.005 ± 0.087 (0.288)	0.279 ± 0.019	0.291 ± 0.031	0.011 ± 0.026 (0.757)	0.374 ± 0.054	0.390 ± 0.056	0.016 ± 0.039 (0.524)
FIB-4 Index	1.370 ± 0.131	1.590 ± 0.305	0.220 ± 0.243 (0.696)	0.864 ± 0.069	0.963 ± 0.104	0.099 ± 0.067 (0.339)	1.206 ± 0.200	1.291 ± 0.180	0.085 ± 0.055 (0.059)
NFS	-0.706 ± 0.143	-0.634 ± 0.192	0.073 ± 0.121 (0.555)	-1.045 ± 0.253	-1.045 ± 0.235	-0.001 ± 0.107 (0.840)	-0.859 ± 0.235	-0.705 ± 0.227	0.154 ± 0.099 (0.133)
FLI score	66.10 ± 4.74	58.37 ± 4.97	-7.73 ± 2.65 (0.008)*	87.98 ± 2.67	82.49 ± 3.56	-5.49 ± 2.29 (0.040)*	71.33 ± 4.98	65.87 ± 5.19	-5.46 ± 2.54 (0.041)*
MRI-PDFF (%)	12.31 ± 1.76	9.34 ± 1.39	-2.98 ± 1.04 (0.007)*	19.51 ± 1.91	17.50 ± 1.76	-2.03 ± 2.00 (0.351)	13.91 ± 1.50	14.02 ± 1.51	0.11 ± 0.91 (0.756)
>30% LFF reduction	13/25 (52%)			3/25 (12%)			2/28 (7.1%)		

Fibrosis scores, elastography, liver enzymes, and lipids

Shearwave elastography and various scores were used to assess possible liver fibrosis of the participants. Mean and median values were calculated for each group of patients at inclusion and at 52 weeks. In all groups, no statistically significant alteration, regarding SWE, FIB-4, APRI, and NFS scores, was observed during treatment. Only notable exception is the reduction of the median SWE value in the Empagliflozin group (*P *= 0.046), but both baseline and end-of-follow-up (EOFU) values were within normal range. Similarly, concerning AST and ALT changes during the follow-up, only the Empagliflozin group exhibited a significant reduction in ALT (P = 0.008), with baseline and EOFU values remaining within the normal range. As far as total cholesterol, LDL, and triglycerides are concerned, no significant reduction was observed in all groups. A small but significant increase in HDL was observed in the Control group (P = 0.034), with no significant difference detected in between-group comparisons. Table 3 showcases further details.

Correlations and binomial logistic regression analysis

Using the Spearman correlation coefficient, a positive correlation between variations of LFF and BMI (*r *= 0.623; *P *= 0.000), HbA1c (*r *= 0.525; *P *= 0.000), FLI score (*r *= 0.582; *P *= 0.000), and triglyceride levels (*r *= 0.259; *P *= 0.022) was detected. Furthermore, variations in LFF correlated significantly with changes in AST and ALT levels, as well as variations in the APRI score and waist and hip circumference. There was also a statistically significant correlation between variations of the APRI, NFS, and FIB-4 scores (Table 4).

**Table 4:** Correlations between study parameters’ variations. ^**^Correlation is significant at the 0.01 level (two-tailed). ^*^Correlation is significant at the 0.05 level (two-tailed). ALT, alanine aminotransferase; APRI, aspartate aminotransferase-to-platelet ratio index; AST, aspartate aminotransferase; BMI, body mass index; FIB-4, Fibrosis-4; FLI, fatty liver index; HbA1c, glycated hemoglobin A1c; HC, hips circumference; HDL, high-density lipoprotein; LDL, low-density lipoprotein; NFS, NAFLD fibrosis score; SWE, shearwave elastography; TC, total cholesterol; TG, triglyceride; WC, waist circumference; Δ, change; NAFLD, nonalcoholic fatty liver disease

Spearman's rho	Δ-LFF	Δ-Bodyweight	Δ-BMI	Δ-FLI	Δ-HBA1c	Age	Δ-TC	Δ-TG	Δ-LDL	Δ-HDL	Δ-AST	Δ-ALT	Δ-SWE	Δ-APRI	Δ-FIB-4	Δ-NFS	Δ-Waist	Δ-Hips
Δ-LFF	1.000	0.623^**^	0.623^**^	0.582^**^	0.525^**^	0.075	0.000	0.259^*^	-0.110	-0.090	0.329^**^	0.451^**^	0.190	0.294^**^	0.080	0.193	0.565^**^	0.527^**^
Δ-Bodyweight	0.623^**^	1.000	0.989^**^	0.636^**^	0.473^**^	-0.037	0.024	0.069	-0.025	-0.197	0.401^**^	0.401^**^	0.298^**^	0.315^**^	0.170	0.202	0.906^**^	0.795^**^
Δ-BMI	0.623^**^	0.989^**^	1.000	0.631^**^	0.447^**^	-0.053	0.008	0.070	-0.039	-0.193	0.430^**^	0.410^**^	0.315^**^	0.346^**^	0.216	0.216	0.906^**^	0.771^**^
Δ-FLI	0.582^**^	0.636^**^	0.631^**^	1.000	0.423^**^	-0.091	0.284^*^	0.514^**^	0.092	-0.221	0.348^**^	0.410^**^	0.321^**^	0.271^*^	0.054	-0.037	0.690^**^	0.537^**^
Δ-HBA1c	0.525^**^	0.473^**^	0.447^**^	0.423^**^	1.000	-0.030	0.053	0.137	-0.092	-0.036	0.354^**^	0.487^**^	0.180	0.286^*^	0.070	0.064	0.386^**^	0.334^**^
AGE	0.075	-0.037	-0.053	-0.091	-0.030	1.000	-0.122	-0.023	-0.172	-0.148	-0.126	-0.115	-0.336^**^	-0.140	-0.165	-0.026	-0.032	-0.052
Δ-TC	0.000	0.024	0.008	0.284^*^	0.053	-0.122	1.000	0.533^**^	0.784^**^	0.308^**^	-0.011	0.016	0.023	-0.039	-0.087	-0.156	0.124	0.020
Δ-TG	0.259^*^	0.069	0.070	0.514^**^	0.137	-0.023	0.533^**^	1.000	0.112	-0.109	0.005	0.036	0.127	-0.035	-0.135	-0.166	0.155	0.058
Δ-LDL	-0.110	-0.025	-0.039	0.092	-0.092	-0.172	0.784^**^	0.112	1.000	0.240^*^	-0.056	0.021	-0.034	-0.033	-0.077	-0.098	0.037	-0.046
Δ-HDL	-0.090	-0.197	-0.193	-0.221	-0.036	-0.148	0.308^**^	-0.109	0.240^*^	1.000	-0.174	-0.115	-0.137	-0.098	-0.016	-0.027	-0.112	-0.290^*^
Δ-AST	0.329^**^	0.401^**^	0.430^**^	0.348^**^	0.354^**^	-0.126	-0.011	0.005	-0.056	-0.174	1.000	0.810^**^	0.351^**^	0.905^**^	0.639^**^	0.175	0.367^**^	0.340^**^
Δ-ALT	0.451^**^	0.401^**^	0.410^**^	0.410^**^	0.487^**^	-0.115	0.016	0.036	0.021	-0.115	0.810^**^	1.000	0.326^**^	0.736^**^	0.316^**^	0.002	0.345^**^	0.309^*^
Δ-SWE	0.190	0.298^**^	0.315^**^	0.321^**^	0.180	-0.336^**^	0.023	0.127	-0.034	-0.137	0.351^**^	0.326^**^	1.000	0.326^**^	0.196	0.070	0.251^*^	0.220
Δ-APRI	0.294^**^	0.315^**^	0.346^**^	0.271^*^	0.286^*^	-0.140	-0.039	-0.035	-0.033	-0.098	0.905^**^	0.736^**^	0.326^**^	1.000	0.819^**^	0.465^**^	0.278^*^	0.263^*^
Δ-FIB-4	0.080	0.170	0.216	0.054	0.070	-0.165	-0.087	-0.135	-0.077	-0.016	0.639^**^	0.316^**^	0.196	0.819^**^	1.000	0.720^**^	0.149	0.127
Δ-NFS	0.193	0.202	0.216	-0.037	0.064	-0.026	-0.156	-0.166	-0.098	-0.027	0.175	0.002	0.070	0.465^**^	0.720^**^	1.000	0.120	0.163
Δ-WC	0.565^**^	0.906^**^	0.906^**^	0.690^**^	0.386^**^	-0.032	0.124	0.155	0.037	-0.112	0.367^**^	0.345^**^	0.251^*^	0.278^*^	0.149	0.120	1.000	0.752^**^
Δ-HC	0.527^**^	0.795^**^	0.771^**^	0.537^**^	0.334^**^	-0.052	0.020	0.058	-0.046	-0.290^*^	0.340^**^	0.309^*^	0.220	0.263^*^	0.127	0.163	0.752^**^	1.000

A backward conditional binomial logistic regression analysis examined which variables predicted improvement of 30% of liver fat content. Apart from the choice of medication, other significant predictors were age, sex, and change in BMI (Table 5).

**Table 5:** Factors related to >30% relative reduction of liver fat content as calculated by MRI-PDFF using binomial logistic regression analysis. ^*^P < 0.05 in bold. CI, confidence interval; MRI-PDFF, magnetic resonance imaging-proton density fat fraction; OR, odds ratio; ΔBMI, change in body mass index

Factor	OR (95% CI)	P
Age	1.223 (1.029-1.454)	0.022*
Sex (male)	26.872 (1.398-516.617)	0.029*
ΔBMI	0.225 (0.084-0.604)	0.003*
Empagliflozin treatment	70.755 (2.974-1683.315)	0.008*

Adverse events

Among patients treated with Dulaglutide, one withdrew from the study due to nausea and vomiting. No other significant adverse effects, such as hypoglycemia or infection, were reported.

## Discussion

In this prospective study, we found that Empagliflozin exhibited statistically significant results regarding liver steatosis improvement, as assessed by MRI-PDFF. Furthermore, a substantial portion of the Empagliflozin treatment arm showed a >30% level of improvement at 52 weeks (13/25, 52%, as opposed to 3/25, 12%, for the Dulaglutide group and 2/28, 7.1%, for the Control group), compared to baseline. This finding could possibly be compatible with fibrosis improvement, as supported by Tamaki et al. [19].

Several methods have been utilized for assessing liver steatosis both in clinical practice and clinical studies. Serological markers, such as APRI score, liver biopsy, controlled attenuation parameter (CAP) score, and MRI-PDFF are the most prominent, with the latter being the most reliable as far as noninvasive methods are concerned.

Previous studies have analyzed the effects of various GLP1 receptor agonists and SGLT2 inhibitors on patients with DM2 and NAFLD. Concerning Liraglutide, Petit et al. showed a statistically significant beneficial effect regarding liver steatosis [20], a finding that was confirmed by Yan et al. [4]. Both used MRI for the participants’ follow-up, while Armstrong et al. reported similar results using liver biopsies [3]. All three of these studies had a follow-up of six months.

Kuchay et al. showed a significant improvement in LFF at three months, as measured by MRI-PDFF, with the use of 10 mg Empagliflozin daily [10]. Chehrehgosha et al. exhibited similar results in patients with DM2 and NAFLD [21], while Taheri et al. did the same with nondiabetic patients with NAFLD [11]. However, both studies used CAP at study entry and six months for assessing liver steatosis.

Concerning Dapagliflozin, Eriksson et al. showed that its combination with omega-3 (n-3) carboxylic acids could reduce liver steatosis compared to placebo in individuals with DM2 and NAFLD [22], as assessed by MRI at three months. After six months of treatment with Dapagliflozin, Kurinami et al. showed a reduction of subcutaneous and visceral fat, utilizing the liver-to-spleen attenuation ratio in abdominal CT scans [23], in subjects with DM2 and NAFLD.

Nishimiya et al., in an uncontrolled study utilizing MRI-PDFF, found a beneficial effect of Canagliflozin on hepatic steatosis, after six months of treatment, but with a very small patient sample [24]. Other studies also support the favorable hepatic effect of Canagliflozin, particularly regarding liver enzymes.

Regarding Dulaglutide, Kuchay et al., using an open-label study format and a sample of similar size to ours, showed significant improvement in LFF in the Dulaglutide group [7]. However, the beneficial effect was detected at six months, while we chose a follow-up period of one year.

Furthermore, Semaglutide is quite promising regarding amelioration of liver steatosis and NASH, as was shown by Newsome et al., in noncirrhotic patients with biopsy-proven NASH in a 72-week follow-up [6]. However, no beneficial effect, regarding inflammation and fibrosis was detected on patients with NASH-related compensated cirrhosis by Loomba et al. [25]. Additionally, promising results were observed by Volpe et al., who monitored patients receiving Semaglutide for a year, utilizing several biochemical markers and scores, as well as ultrasonography [26].

However, there is a scarcity of studies examining Dulaglutide or comparing agents from the SGLT2-is and GLP1-ras families, regarding their effect on liver steatosis. Additionally, most studies pursue a short follow-up period of three to six months, which could, in some cases, reveal short-lived trends that do not significantly benefit patients in the long run.

Mechanisms of action resulting in improvement of liver steatosis are probably various but not fully understood. It is known that SGLT2 inhibitors lower bodyweight, through visceral and subcutaneous adipose tissue reduction, facilitated by a shift in substrate utilization towards lipids, and urinary loss of glucose [9,27]. Their effects are multisystemic, including, but not limited to, anti-inflammatory, cardioprotective, and nephroprotective benefits [9,14]. On the same note, GLP1 receptor agonists exhibit multiple pathways of action, displaying multifaceted effects, incorporating HbA1c reduction, increased satiety, calorie intake and bodyweight reduction [28], cardiorenal protection [12,13], and even neuroprotective properties [29], apart from their beneficial effect in NASH and liver steatosis [29,30]. As far as the latter is concerned, the effects of GLP1 receptor agonists include, among others, improvement of hepatocyte mitochondrial function, mitigation of adipose tissue lipotoxicity, increased perilipin phosphorylation, and downregulation of various pro-inflammatory cytokines, such as interleukin-6, tumor necrosis factor-a, and monocyte chemotactic protein 1 [30].

This study has some limitations. First, the sample was not large, participants were mostly male, and the study design was open-label. However, despite the relatively small size, statistically significant results were obtained. Second, there was some degree of heterogeneity between groups, with the Empagliflozin group having the greatest mean age, and the Dulaglutide group having the greatest mean BMI and baseline LFF, as it had been common practice to prescribe GLP1-ras for obese diabetics. Finally, the COVID-19 pandemic hindered the follow-up of the study's participants to some degree. On the other hand, this study was prospective, and both MRI and elastography data were analyzed by the same researchers, who were blinded to the patients' treatment arm. Nevertheless, despite difficulties imposed by lockdowns and social distancing, a follow-up of 52 weeks was successfully completed by most participants in our study, allowing us to conclude that Empagliflozin is more beneficial for diabetics with NAFLD when compared with Dulaglutide.

## Conclusions

In conclusion, this is the first prospective controlled study comparing Dulaglutide with Empagliflozin regarding their effect on liver steatosis, as assessed by MRI-PDFF and various other biomarkers and scores. Empagliflozin proved to be more effective in reducing LFF. However, considering the observational nature of the study and its inclusion of entirely Caucasian subjects, further and larger studies comparing agents of the GLP1 receptor agonists' and SGLT2 inhibitors' families, and possibly various combinations of them, are needed to allow clinicians to tailor treatment according to their individual patients' needs. There is a new era upon us regarding the treatment of NAFLD and DM2. In the face of increasing worldwide demand and incurring periodic shortages of some of these drugs, the necessity of such trials must be emphatically highlighted.
